# Supplementary material for: Quantum-enhanced second harmonic generation beyond the photon pairs regime
Source: Sci Adv. 2025 Jul 4;11(27):eadw4820. doi: 10.1126/sciadv.adw4820 (PMC13155536; doi:10.1126/sciadv.adw4820)
Supplement: Supplementary file 1 — Supplementary Text Fig. S1 [file sciadv.adw4820_sm.pdf]

Science Advances
AAAS

Supplementary Materials for

Quantum-enhanced second harmonic generation beyond the photon pairs regime

Thomas Dickinson *et al.*

Corresponding author: Lucia Caspani, lucia.caspani@uninsubria.it

Sci. Adv. **11**, eadw4820 (2025)
DOI: 10.1126/sciadv.adw4820

This PDF file includes:

Supplementary Text
Fig. S1

Supplementary Text

Generation of the theory curves

The theoretical model for SHG driven by the SV, addressing the quantum to classical comparison between the SV illumination and a coherent classical illumination, was proposed and developed in Ref. *(35)*. For the benefit of the reader, we summarize in the following some formulas used to produce the theory curves in the main manuscript.

The model in *(35)* assumes a PDC pump with a Gaussian amplitude profile: $A_p(\vec{\xi}) = \exp(-(x^2+y^2)/D_p^2)\exp(-t^2/\tau_p^2)$, where D_p and τ_p are the spatial and temporal widths of the Gaussian pump field profile. In the following, we use the shorthand notation for the space-time coordinate $\vec{\xi} = (x,y,t) = (\vec{r},t)$. Using a fully spatiotemporal model for the nonlinear interactions in the PDC and SHG crystals, as well for the propagation between the two crystals, the theory developed in Ref. *(35)* allows to evaluate the output quantum fields, and thus the relevant quantities, such as the photon flux density and number of photons per pulse for the SV and SH coherent and incoherent components generated by the SV, as well as the SH signal generated by the classical coherent input.

In particular, the number of SV photons per pulse, N_{SV}, can be calculated by integrating the SV photon flux density in Eq. (29) of Ref. *(35)* [see also Eq. (S34) of Ref. *(35)*]:

$$N_{SV} = \int d_3\xi F_{coh}(\vec{\xi}) \cdot \int \frac{d_3\vec{w}}{(2\pi)^3}|V(\vec{w})|^2, \qquad \text{Eq. (S1)}$$

where $\vec{w} = (q_x, q_y, \Omega)$ is the Fourier conjugate variable of $\vec{\xi}$, q_i is the transverse momentum ($i = x, y$) and Ω is the frequency offset from the carriers, with $\vec{w}\cdot\vec{\xi} = xq_x + yq_y - \Omega t$. The function

$$F_{coh}(\vec{\xi}) = \frac{\sinh^2[2gA_p(\vec{\xi})]}{\sinh(2g)} \qquad \text{Eq. (S2)}$$

describes the photon flux density profile of the SV field, where

$$g = \Lambda\sqrt{N_P} = 2\, l_c d_{\text{eff}}\sqrt{\frac{\hbar\omega_P\omega_{SV}^2}{2\varepsilon_0 n_P\, n_{SV}^2 c^3\, V_P}}\sqrt{N_P} \qquad \text{Eq. (S3)}$$

is the parametric gain, being l_c the crystal length, V_P the pump spatiotemporal volume (in m^2 s), and N_P the number of photons of the pump pulse. Note that in *(35)* the parametric gain is defined as $g = \chi l_c|\alpha_P|$, where $|\alpha_P|^2 = N_P/V_P$ is the peak photon flux density of the pump and $\chi = 2\, d_{\text{eff}}\sqrt{\frac{\hbar\omega_P\omega_{SV}^2}{2\varepsilon_0 n_P\, n_{SV}^2 c^3}}$.

As detailed in Ref. *(40)*, $F_{coh}(\vec{\xi})$ coincides with the pump photon flux density profile $|A_p(\vec{\xi})|^2$ at low gains ($g \ll 1$), while it shrinks as $F_{coh}(\vec{\xi}) \sim \sqrt{\tanh g/g}$ at increasing gain.
The second term at the r.h.s. of Eq. (S1) includes the SV one-photon probability distribution in the Fourier space

$$|V(\vec{w})|^2 = \left|g\frac{\sinh\Gamma(\vec{w})}{\Gamma(\vec{w})}\right|^2,$$

which is strongly peaked in the regions of the Fourier space where phase-matching is realized because

$$\Gamma(\vec{w}) = \sqrt{g^2 - \frac{[\mathcal{D}(\vec{w}, -\vec{w}) l_c]^2}{4}}$$

is a "weighted" gain function including the effect of the phase mismatch $\mathcal{D}(\vec{w}, -\vec{w}) = k_{SV,z}(\vec{w}) + k_{SV,z}(-\vec{w}) + k_{P,z}(0)$, being $k_{i,z}$ the longitudinal wave vector of the i-th field.

We note that these results were obtained under a "quasi-stationary" approximation *(35,40,41)*, which assumes that both the pump driving the PDC and the SV beams have a large cross-section and temporal duration, much larger than the characteristic spatial and temporal widths of the entanglement volume. In addition, it assumes that the spatial walk-off between the pump and SV beams is not relevant, which is the case for our large pump (1.5 mm intensity FWHM), and does not include the effects of GVM.

As mentioned in the main manuscript, the quantity $\sinh^2 g = \langle n \rangle_m$ represents the number of SV photons per mode. Following the theoretical model, N_{SV} is evaluated by numerically calculating the integrals in Eq. (S1) for varying parametric gain, g. The plot N_{SV} vs g is then transformed in N_{SV} vs N_P using Eq. (S3), resulting into the theoretical curve in Fig. 2A in the main manuscript (red solid curve).

From N_{SV} we then extract the number of modes K_m as:

$$K_m = \frac{N_{SV}}{\langle n \rangle_m} = \frac{\int d_3\vec{\xi} F_{coh}(\vec{\xi}) \cdot \int \frac{d_3\vec{w}}{(2\pi)^3} |V(\vec{w})|^2}{\sinh^2(g)}. \qquad \text{Eq. (S4)}$$

The plot of Eq. (S4), expressed as a function of N_P rather than g, is shown in the inset of Fig. 2B in the main manuscript, and K_m is the function used to evaluate $\langle n \rangle_m$ from the measured value N_{SV}.
Combining Eqs. (S1), (S2) and (S3), we numerically evaluated the width of the SV photon flux density profile, $F_{coh}(\vec{\xi})$, as a function of N_{SV}. The result is the theoretical curve reported in Fig. 2B (red solid line).

Similarly, the theoretical model allows to evaluate the number of SH photons per pulse, for the coherent and incoherent components, N_{SH}^{E} and N_{SH}^{inc} [Eqs. (26) and (27) in Ref. *(35)*], respectively, as well as for the classical comparison [N_{SH}^{CL}, Eq. (33) in Ref. *(35)*]. We have used these expressions to calculate the theoretical curves in Fig. 4: the solid green curve represents N_{SH}^{E}/N_{SH}^{CL} while the dashed red curve represents $\left(N_{SH}^{E} + N_{SH}^{inc}\right)/N_{SH}^{CL}$.

Theoretical analysis of the impact of losses

We have used the theory discussed in the main text and fully reported in (*35*) to model the effect of losses on the SV field and compared it to the experimental results.
The theoretical results obtained for $\langle n_m \rangle \leq 1$ are shown in Fig. S1A overlapped with the experimental data points. Here, we have considered a $d'_{\text{eff}} = 1.65$ pm/V and fitted the model to the experimental data. The fitting parameter is imposed on the number of SH photons and hence is a constant multiplication parameter. The experimental values for the number of SH photons are 35% smaller than the theoretical predictions. We note that the fit with the constant scaling

parameter works well for the no-losses and 30% losses conditions, whereas it over-estimates the SH photons for the 50% losses case. We note that these values only include absorption losses (measured for each filter), but a non-negligible effect is also expected from the dispersion introduced by each neutral filter. A quantitative comparison with the theory is therefore not straightforward, but the model recreates very well the qualitative behavior observed in the experiments.

We also extended the theoretical model at higher SV average number of photons per mode. The theoretical results are shown in Fig. S1B without any correction. They qualitatively reproduce the experimental trend (Fig. 3B in the main text). Indeed, crossing points between the three cases occur, albeit at different values with respect to those measured in the experiments.

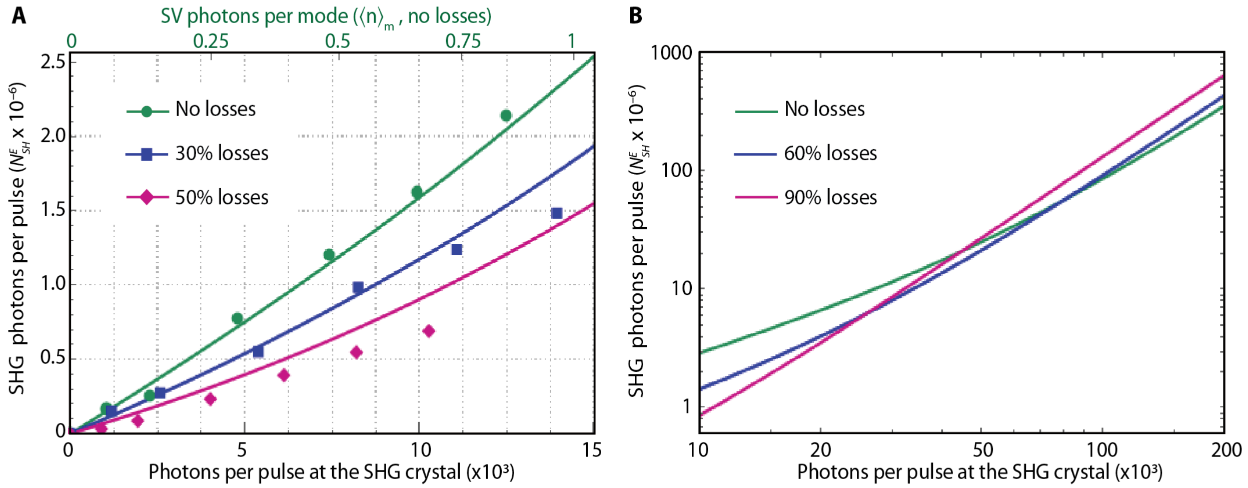

Fig. S1 A. Results of the theoretical model of the eSHG processes driven by SV (green) and loss-spoiled SV states (solid curve). The points are experimental values. The theory quantitatively predicts the experimental findings assuming 35% losses and a reduced effective nonlinearity. **B.** Theoretical prediction of the impact of losses on the eSHG counts at higher SV intensities. As seen in the experiments, the efficiency of the higher loss cases overcome those of the lower loss cases, due to the change in SV pulse intensities consequent of its gain-dependent profile.
